# Epigenetic dysregulation in acute myeloid leukemia ^☆^

**DOI:** 10.1053/j.seminhematol.2025.06.003

**Published:** 2025-06-19

**Authors:** Jens Schrezenmeier, B.J.P. Huntly

**Affiliations:** a Cambridge Stem Cell Institute, University of Cambridge, Cambridge, United Kingdom; b Department of Haematology, University of Cambridge, Cambridge, United Kingdom; c Department of Haematology, Cambridge University Hospitals NHS Foundation Trust, Cambridge, United Kingdom

## Abstract

Acute myeloid leukemia (AML) is an aggressive hematologic malignancy defined by the clonal expansion of undifferentiated myeloid blasts with a block in differentiation and aberrant self-renewal. While recurrent genomic mutations are well-documented in AML, epigenetic dysregulation has emerged as an equally pivotal driver of leukemogenesis, a notion corroborated by the frequent recurrence of mutations in epigenetic regulators. Leukemic cells exhibit pervasive epigenetic alterations—including abnormal DNA methylation patterns, dysregulated histone modification, disrupted chromatin architecture and RNA-based regulatory mechanisms —which collectively rewire gene expression programs. These changes silence key differentiation genes and sustain self-renewal pathways, enforcing the developmental arrest and hyper-proliferation that are the hallmarks of AML. Importantly, epigenetic aberrations in AML are not merely downstream consequences of genetic lesions but actively contribute to the malignant phenotype. Somatic mutations frequently target epigenetic regulators (for example, DNA methyltransferases or histone modifiers), and these lesions cooperate with other genetic alterations to initiate and maintain the leukemic clone. Together, these insights highlight epigenetic dysregulation as a central mechanism in AML pathogenesis.

## Introduction

Acute myeloid leukemia (AML) is characterized by uncontrolled, clonal proliferation of myeloid precursor cells with impaired differentiation, dominance over normal haematopoiesis and subsequent symptoms of bone marrow failure. While AML has classically been defined by recurrent genetic abnormalities such as chromosomal translocations and gene point mutations, it is now evident that epigenetic deregulation plays an equally critical role in its pathogenesis. Epigenetics refers to heritable changes in gene expression that do not involve changes to the underlying DNA or mRNA sequence, and these changes include alterations in DNA methylation, histone modification, higher-order chromatin structure as well as RNA modifications, so-called “epitranscriptomics.” Abnormal patterns in these epigenetic marks are pervasive in AML and are consequent on and often cooperate with genetic lesions to drive leukemogenesis [1].

Comprehensive genomic studies have revealed that nearly half of AML cases harbor mutations in genes regulating DNA methylation (eg DNMT3A, TET2, IDH1, IDH2) and about one-third have mutations in genes that directly or indirectly modify chromatin (eg KMT2A/MLL, ASXL1, EZH2) [2].

These mutations highlight a direct link between genetic and epigenetic aberrations: they disrupt the enzymes or cofactors that establish and maintain normal epigenetic landscapes, leading to widespread changes in gene expression programs crucial for normal hematopoietic differentiation and facilitating aberrant self-renewal [3]

Epigenetic deregulation in AML manifests in several forms. Global DNA methylation profiles in AML are commonly skewed, with regions of aberrant hypermethylation at tumor suppressor gene promoters and focal hypomethylation at certain repetitive elements or enhancers [4]. Similarly, histone modifications that facilitate chromatin accessibility and gene transcription are altered; for example, leukemic blasts can exhibit inappropriate gain or loss of repressive histone marks at key differentiation genes [5] In addition, higher-order chromatin architecture may be perturbed through mutations in Cohesin complex members, further disturbing gene regulation [6,7]. RNA modifiers have also recently been demonstrated to be involved in the epigenetic circuitry of AML, as they can reinforce malignant gene expression patterns [8,9].

Understanding these layers of epigenetic dysregulation is vital because, unlike fixed DNA mutations, epigenetic changes are potentially reversible [10], and thus more amenable to therapeutic intervention. This plasticity therefore provides a compelling rationale for epigenetic therapies. The following sections will detail the major modes of epigenetic deregulation in AML – related to: DNA methylation alterations, aberrant histone modification, chromatin remodeling defects and alterations in RNA modifications – and will further discuss how each contributes to leukemogenesis. We will then briefly review the impact of a better understanding of disease biology to improve epigenetic treatment strategies and briefly discuss emerging therapies.

### DNA methylation abnormalities in AML

DNA methylation, the addition of methyl groups to cytosine bases (primarily the 5' carbon of Cytosine at CpG dinucleotides), is a key epigenetic mechanism governing gene expression [11]. In healthy hematopoietic cells, DNA methylation patterns tightly regulate lineage-specific gene transcription [12]. In AML, however, DNA methylation landscapes are profoundly altered [13] Aberrant DNA hypermethylation at promoter CpG islands of critical genes is commonly observed, leading to their transcriptional silencing [14]. Concurrently, some genomic regions become hypomethylated, which can activate oncogenes or endogenous genomic repeats [15]. These irregular methylation patterns can be a direct consequence of mutations in enzymes that write, read, or erase DNA methylation marks [16], although their correlation with altered gene expression patterns remains poorly explained.

#### DNMT3A mutations

**Fig. 1:**
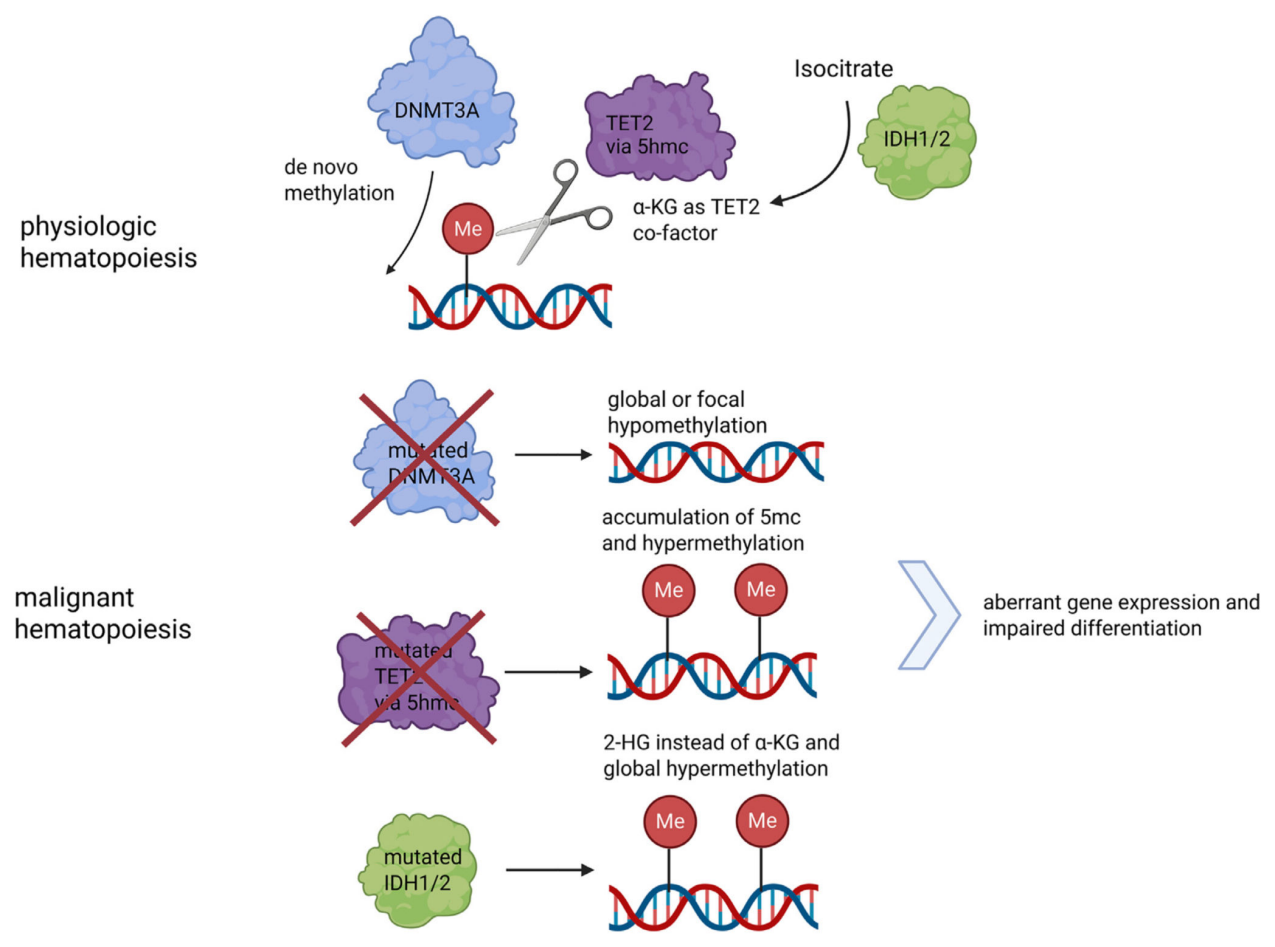
Normal State (top panel): DNMT3A, TET2, and IDH1/2 cooperate to maintain balanced DNA methylation in their unmutated states. Mutated Genes (panels below): mutated DNMT3A causes loss of its de novo methylation function leading to regions of hypomethylation or global hypomethylation depending on the mutation type. Mutated TET2 loss-of-function leads to global hypermethylation (since 5mC is not effectively converted to 5hmC). IDH1/2 mutations produce 2-HG that inhibits TET2, also resulting in hypermethylation. Downstream effects are dysregulated methylation patterns, block of normal differentiation programs and enhanced self-renewal, driving leukemic transformation are the consequence. BCAT1 overexpression (not shown): Even without an IDH mutation, high BCAT1 depletes *α*-KG, mimicking IDH-mutant effects (TET2 inhibition and hypermethylation). All these pathways converge on aberrant methylation that disrupts normal hematopoietic differentiation and fuels AML development.

One of the most frequent mutations in AML occurs in the DNMT3A gene. DNMT3A is a de novo DNA methyltransferase with a multi-domain structure. DNMT3A mutations are found in roughly 20%-25% of adult AML patients, often those with intermediate-risk cytogenetics [11,16]. The most common mutation is an Arginine substitution at codon 882 in the DNMT3A enzyme which occurs in around 60% of cases in AML and disrupts the oligomerization interface [17]. DNMT3A R882 mutations typically result in hypomorphic methylation activity, leading to focal hypomethylation at specific gene regions and are also the most common mutation that contributes to the pre-leukemic state of clonal hematopoiesis [18,19]. Consistent with this, AML cases with DNMT3A mutations often show broad shifts in methylation, and in experimental models DNMT3A disruption propagates self-renewal of hematopoietic stem cells at the expense of differentiation (Fig. 1) [20,21]. It was also demonstrated that DNMT3A-mutated HSC clones can survive induction chemotherapy and remain present at remission [22,23]. Notably, such DNMT3A-mutated pre-leukemic cells were found in patients whose bulk leukemia cells also carried NPM1 and FLT3-ITD mutations, implying DNMT3A lesions arose earlier and persisted after the transient remission of the NPM1/FLT3-ITD-mutant leukemic clone.

Recent studies have also begun to elucidate distinct mechanisms by which various non-R882 DNMT3A mutations contribute to leukemogenesis. A systematic functional profiling of 253 DNMT3A variants revealed that ~74% of patient-derived mutations cause loss of DNA methyltransferase activity, and about half of these loss-of-function variants also exhibit markedly reduced protein stability [24]. These unstable DNMT3A mutants were shown to undergo proteasomal degradation lowering the cellular level of DNMT3A protein. As a class, such DNMT3A-destabilizing mutations are associated with greater clonal expansion of HSCs and higher likelihood of AML development, albeit less than the common stable variant R882-mutations. These data suggest that unstable mutations confer a strong fitness advantage to mutant clones. Importantly, the methylomes of DNMT3A-destabilizing variants differ from R882-mutated variants, with patterns more similar to WT DNMT3A patients. Mechanistically, unlike DNMT3AR882 mutants which enforce specific hypomethylation patterns, DNMT3A destabilizing mutants appear to create increased intratumor DNA methylation heterogeneity and transcriptional variance [18].

#### TET2 mutations

Another major regulator of DNA-methylation mutated in AML is the TET2 (ten-eleven translocation 2) gene, occurring in approximately 10-20% of cases (with higher frequencies in older patients and secondary AML) [25]. TET2 is a 2-oxoglutarate (*α*-ketoglutarate)-dependent dioxygenase that catalyzes iterative oxidation of 5-methylcytosine (5mC) in DNA to yield 5-hydroxymethylcytosine (5hmC), 5-formylcytosine, and 5-carboxylcytosine [26]. In hematopoietic progenitors, TET2 is highly expressed and enriched at active enhancers, where 5hmC accumulates as an intermediate of active DNA demethylation. TET2-mediated cytosine oxidation at these regulatory regions helps maintain an open chromatin state that facilitates transcription factor binding and lineage-specific gene activation. Loss of TET2 function reduces 5hmC and leads to aberrant hypermethylation of enhancers, which diminishes enhancer accessibility and perturbs the expression of genes driving differentiation (Fig. 1) [27]. TET2-mutant AML exhibits global hypermethylation and impaired expression of genes involved in myeloid differentiation [26].

Beyond this canonical role in DNA demethylation, an RNA-dependent mechanism by which TET2 loss may contribute to leukemogenesis was recently described. This study revealed that TET2 regulates chromatin state through RNA-mediated control of histone modifications [28]. Specifically, TET2 can oxidize 5-methylcytosine on chromatin-associated RNA. Methylated RNA (m5C) is recognized by the methyl-CpG-binding protein MBD6, which recruits a deubiquitinase complex to remove monoubiquitin from histone H2A at Lys119 (H2AK119ub) – a repressive mark – thereby opening local chromatin [28]. TET2 normally antagonizes this process by oxidizing m5C in the RNA, preventing MBD6 from binding. Consequently, when TET2 is lost, m5C RNA persists and MBD6 activity is unrestrained, leading to globally decreased H2A ubiquitination, a more open chromatin architecture, and widespread transcriptional upregulation in progenitor cells [28].

#### IDH mutations

Mutations in the metabolic enzymes IDH1 and IDH2 provide a striking link between metabolism and epigenetics in AML. The metabolic enzymes IDH1 and IDH2 normally catalyze the oxidative decarboxylation of isocitrate to *α*-ketoglutarate (*α*-KG) [29], in the cytoplasm and mitochondria, respectively. Through production of *α*-KG, wild-type IDH1/2 supply substrates to numerous *α*-KG-dependent dioxygenases (eg, TET DNA hydroxylases and JmjC-domain histone demethylases) that modulate the epigenome. IDH1/2 mutations occur in roughly 15%-20% of AML (with IDH1 mutated in ~6%-10% and IDH2 in ~12% of patients) and are neomorphic, endowing the enzyme with a new activity [30]. Mutant IDH1/2 further process *α*-ketoglutarate (*α*-KG) to the oncometabolite 2-hydroxyglutarate (2HG). 2HG is structurally similar to *α*-KG and acts as a competitive inhibitor of *α*-KG-dependent dioxygenases, including TET2 and certain histone demethylases [29]. IDH1/2-mutant AML cells accumulate 2HG, which impairs TET2 function and leads to a build-up of DNA methylation marks leading to a block in differentiation. Hypermethylation at promoters of myeloid differentiation genes prevents their expression, trapping cells in an immature, proliferative state (Fig. 1) [31], that is pro-leukemogenic. The discovery of this mechanism demonstrated how a single metabolic mutation can reprogram the epigenome and induce leukemia. IDH1/2 and TET2 mutations converge on similar epigenetic mechanisms and are seldom found together [1]. However, loss-of-function TET2 variants are common in clonal hematopoiesis and predominate in AML of older adults, while IDH1/2 mutations are uncommon in CHIP, yet when such clones do arise they carry a markedly elevated risk of progression to overt leukemia [32].

#### IDH-like epigenetic phenotypes

Beyond genetic mutations, metabolic perturbations can also induce IDH-like epigenetic reprogramming. A striking example relates to the branched-chain amino acid transaminase BCAT1, which was found to be upregulated in some IDH-wildtype AML cases. BCAT1 depletes intracellular *α*-ketoglutarate, mimicking the effect of mutant IDH [33]. BCAT1high/IDH WT AML cells undergo extensive DNA hypermethylation through TET inhibition, closely recapitulating the IDH-mutant methylation profile. Clinically, BCAT1high IDH-wildtype leukemias demonstrated the same hypermethylation signature and were associated with a stem-cell–like gene expression program [33]. This finding links altered cancer metabolism to epigenetics, highlighting that IDH-mutant and IDH-like AML share a convergent epigenetic program.

In summary, aberrant DNA methylation is a pervasive feature of AML, albeit that there is not a single pathological pattern of DNA alteration. Methylation changes silence critical regulators of differentiation and allow expansion of leukemic clones by cell autonomous and non-cell autonomous mechanisms. However, because DNA methylation is reversible, it represents a compelling therapeutic target – a concept that has been validated by the clinical activity of hypomethylating agents in myeloid malignancies as well as the mutation-specific inhibition of neomorphic enzyme function in IDH1- and IDH2-mutated AML [34–37].

### Dysregulation of histone modification and chromatin remodeling

Gene expression is profoundly influenced by histone modifications – chemical marks on histone proteins around which DNA is wrapped. These post-translational modifications (that include methylation, acetylation, ubiquitination, etc.) alter chromatin structure, histone interaction with DNA and recruit effector proteins, thereby regulating transcriptional activity. In AML, the machinery that writes, erases, or interprets histone marks is frequently disrupted, leading to chromatin dysregulation and inappropriate gene expression programs. Several recurrent mutations in AML directly affect histone modifiers or chromatin regulators, underscoring the centrality of histone-level epigenetic deregulation in leukemia.

#### MLL mediated chromatin deregulation

A prominent example of epigenetic dysregulation in AML, that has greatly educated the field, is provided by leukaemias with MLL (Mixed Lineage Leukaemia, also known as KMT2A) gene rearrangements. MLL rearrangements (typically 11q23 translocations) create chimeric oncoproteins, that fuse the N-terminus of MLL to 1 of over 60-100 possible partners (eg) [38].

**Fig. 2:**
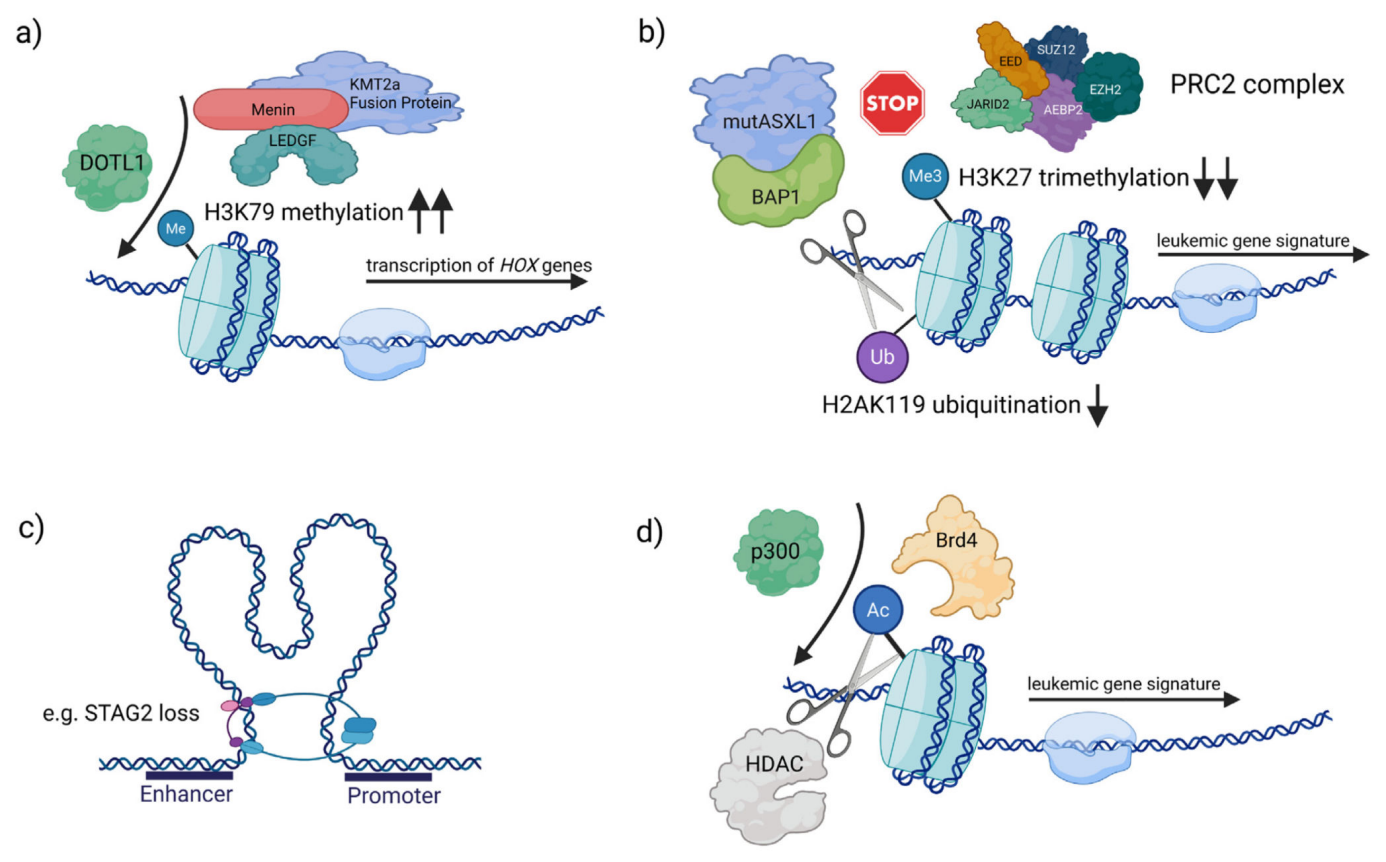
(A) MLL Fusion oncoproteins engage Menin, LEDGF, and DOT1L to cause abnormal H3K79 methylation (B) ASXL1/PRC2 dysfunction caused by loss of function mutations of ASXL1 lead to reduced H3K27me3 levels in addition to the continued deubiquitination activity of the mutASXL1/BAP1 complex and a loss of repressive gene control (C) Cohesin/Chromatin remodeling alterations cause disrupted genome topology, affeccting enhancer–promoter interactions. (D) Histone acetylation imbalance by corrupted acetyl writer (eg p300), reader (eg BRD4) and deacteylase (HDACs) activity. All these changes converge on the aberrant activation of key leukemogenic programs (eg, HOXA9/MEIS1) that promote self-renewal while inhibiting differentiation.

These fusion partners are often components of transcriptional elongation complexes. Seventy percent involve the AF9, AF4 and ENL proteins, components of or recruiters of the super-elongation and DOT1L containing complexes that regulate transcriptional elongation [39], and their recruitment fundamentally alters histone modifications at critical gene loci. Wild-type MLL is a histone H3 lysine 4 (H3K4) methyltransferase important for normal HOXA-cluster gene activation during haematopoiesis (Fig. 2) [40]. In contrast, MLL fusion proteins lack the C-terminal SET domain responsible for H3K4 methylation and instead aberrantly recruit alternative chromatin-modifying machinery [41], including the DOT1L complex (disruptor of telomeric silencing 1-like), the sole enzyme that methylates histone H3 lysine 79 (H3K79) [42]. Through this mechanism, MLL fusions mistarget DOT1L to MLL target genes, leading to ectopic H3K79 methylation and enforced expression of leukemogenic transcriptional programs [40,43]. In addition, another fusion partner ELL (RNA polymerase II (Pol II) elongation factor) suggested RNA-PolII elongation as a mechanism of leukaemia-specific transcription and proteomic studies confirmed several MLL translocation partners in a transcriptional super elongation complex (SEC) [39]. Developmental genes such as HOXA9 (often with its cofactor MEIS1) are key targets with their regulatory elements decorated with activating marks and their gene bodies with H3K79 methylation driving persistent high-level expression [44,45].

A critical cofactor in this process is Menin, a nuclear scaffold protein encoded by MEN1. Menin binds directly to the N-terminal fragment of MLL present in all MLL fusion proteins [46], tethering MLL oncoproteins to chromatin. It forms a complex with chromatin adaptor LEDGF, which in turn binds H3K36me2, anchoring the MLL fusion to specific genomic loci such as HOXA9/MEIS1 promoters (Fig. 2) [47,48]. Importantly, Menin also helps recruit DOT1L to these loci, thereby coupling the MLL fusion to the enzymatic deposition of H3K79 methylation [41]. The net effect is an epigenetic environment that maintains leukemic stem cell self-renewal and blocks differentiation. The HOXA9/MEIS1-driven programme enforced by MLL–Menin–DOT1L complexes confers stem cell-like properties [45].

Consistently, loss-of-function studies have shown that these epigenetic collaborators are indispensable: Dot1l deletion abrogates MLL-AF9 leukemogenesis in models, and Menin is likewise essential to sustain the HOX/MEIS transcriptional programme in MLL-rearranged cells [49]. This mechanistic dependency has spurred targeted therapeutic approaches against the MLL fusion complex. One strategy is to inhibit DOT1L and thereby reverse the aberrant H3K79 methylation. The first-in-class DOT1L inhibitor Pinometostat (EPZ-5676) provided proof-of-concept: treatment led to reduced H3K79 methylation and downregulation of HOX/MEIS1 expression in MLL-rearranged leukemia [50]. Clinically, Pinometostat showed only modest monotherapy activity [51]. A more recent and promising approach is to disrupt the Menin–MLL interaction. Small-molecule Menin inhibitors (eg revumenib/SNDX-5613 and ziftomenib) bind menin’s pocket, preventing it from engaging MLL fusion proteins on chromatin [52]. Early clinical trials of menin inhibitors have reported encouraging results in MLL-rearranged and NPM1-mutated AML [53,54]. These inhibitors will be covered in more detail in a companion review article of this special issue (S Armstrong and colleagues).

Similarly, residual mutant NPM1c remaining within the nucleoplasm has been demonstrated to bind directly to chromatin at a number of loci, including at HOXA/MEIS1 loci in cooperation with the Menin-MLL1–histone methyltransferase complex, sustaining their transcriptional activation [55]. AML cells with NPM1c are consequently sensitive to Menin–MLL1 inhibitors that disrupt this interaction. Likewise, NUP98 fusion oncoproteins and the recently described UBTF (Upstream binding transcription factor) tandem duplications depend on the Menin–MLL1 complex to maintain a HOX/MEIS-centered gene expression programme; pharmacologic Menin–MLL1 blockade in NUP98-rearranged or UBTF-mutated AML displaces MLL1 and either the fusion or UBTF from target chromatin, downregulating HOXA/MEIS1 and inducing myeloid differentiation [56,57]. These findings underscore a unifying theme that diverse AML oncogenic lesions hijack MLL1-mediated histone modifications to aberrantly activate developmental genes.

#### Non-MLL mediated chromatin deregulation and HOX activation

In addition to MLL1 complexes, other chromatin regulators reinforce the HOX/MEIS transcriptional programme. A recent study showed that a point mutation in the acetyl-lysine reader ENL (MLLT1) endows it with the ability to form discrete phase-separated condensates at the HOXA cluster and MEIS1 genomic loci, driving their overexpression and rapidly inducing AML in vivo [58]. Disrupting ENL’s acetyl-binding function—either by specific mutagenesis or a small-molecule YEATS domain inhibitor—was sufficient to dissolve these condensates and impair ENL-driven leukaemogenesis [58]. In addition, the chromatin-reading coactivator SGF29 (a Tudor-domain subunit of the SAGA complex that binds to H3K4me2/3), has been identified as a critical dependency maintaining AML stem cell programmes [59]. CRISPR screens revealed that SGF29 is required to sustain the transcription of key AML oncogenes, and SGF29 loss markedly impairs leukaemic self-renewal across multiple AML subtypes [59]. Together, these insights highlight that aberrant “reader” proteins of histone marks (ENL, SGF29) can drive or maintain the transcriptional circuitry of AML, especially the HOXA network, in the absence of traditional mutations in writer enzymes.

#### ASXL1 and EZH2 mutations, altered PRC2 complex function, H3K27 and others

The ASXL1 (Additional Sex Combs-Like 1) gene is also a commonly mutated chromatin regulator in AML, and is, found in ~5-10% of AML cases (more frequently in the elderly and in secondary AML/MDS). It is the mammalian homolog of Drosophila Asx and can both activate and repress developmental genes like HOX through interactions with Polycomb (PcG) and Trithorax (TrxG) complexes [60]. In haematopoietic cells, wild-type ASXL1 associates with PRC2 members (EZH2, EED, SUZ12) to facilitate H3K27me3 deposition and gene silencing [61]. Concurrently, ASXL1 interacts with the deubiquitinase BAP1 (forming the PR-DUB complex) to remove repressive H2A ubiquitin (H2AK119ub) marks (Fig. 2) [62]. Through these dual activities, ASXL1 dynamically helps maintain proper chromatin states – for example, ensuring that inappropriate HOX gene expression is suppressed outside of developmental contexts– thereby preserving the balance between haematopoietic self-renewal and differentiation. Loss-of-function mutations in ASXL1 lead to a reduction of H3K27me3, derepression of PRC2 target genes, and promotion of leukemogenesis [63]. In addition, loss-of-function mutants can still aberrantly interact with BAP1, leading to enhanced H2A deubiquitination and reduced global H3K27me3 (Fig. 2). This epigenetic dysregulation causes widespread changes in chromatin accessibility, including aberrant opening of chromatin at stemness-related and HOXA gene loci [64]

EZH2, the catalytic subunit of PRC2 responsible for H3K27 trimethylation on the other hand, has a complex stage-specific role in AML pathogenesis. Somatic EZH2 mutations are uncommon, present in only ~1%-2% of de-novo AML but rising to ~10% in secondary or therapy-related cases and patients with aberrations of chromosome 7 [65,66]. It could be demonstrated that

EZH2 exhibits opposing functions: acting as a tumor suppressor during leukaemic initiation while serving as an oncogenic driver in AML maintenance [67]. Loss of EZH2 (and its repressive H3K27me3 marks) in early disease derepresses a subset of normally silenced genes – including HOXA gene clusters and other stemness-associated programs – that accelerate leukaemogenesis [67]. Conversely, established AML cells rely on EZH2 to repress differentiation-inducing or tumour-suppressive pathways, thereby preserving a leukemic stem-like state; accordingly, EZH2 inactivation downregulates AML maintenance programs without reactivating HOX loci. Consistent with this duality it was shown that diminished EZH2 expression in AML leads to HOXA9/HOXB7 upregulation and enhanced self-renewal, conferring therapy resistance and poor prognosis [68].

In addition to PRC2 loss-of-function, lesions in the H3K27 methylation pathway can also promote AML by disrupting the balance of histone modifications. The UTX (KDM6A) demethylase – recurrently mutated or deleted in ~3% of AML [1] – normally removes H3K27me3 in the stepwise process of gene activation. However, intriguingly, UTX-loss rewires the chromatin landscape through non-catalytic effects. In a murine AML model, Utx deletion led to only minor increases in global H3K27me3 but pronounced bidirectional shifts in H3K27ac and chromatin accessibility [69]; a predominant loss of H3K4me1 modifications and alterations in ETS and GATA-factor TF binding [69]. Proteomic analyses linked these changes to the role of UTX to coordinate the binding of ATP-dependent chromatin remodelers and the COMPASS H3K4-methylation complex to generate and activate critical enhancers.

To summarize, H3K27-modifying enzymes are central to AML pathogenesis: both loss of silencing and a failure to initiate activation (ASXL1/PRC2 dysfunction or UTX mutation, respectively) tips the balance toward pro-leukaemic gene expression programmes, both by directly de-repressing stemness genes and/or by indirectly reprogramming enhancer–promoter interactions.

#### Histone acetylation

Disruption of the balance of histone acetylation is another important aspect in AML epigenetics. Histone acetylation (eg at H3K27 or H3K9/14) is generally linked to open chromatin and active transcription. Net acetylation levels reflect the opposing actions of histone acetyltransferases (HATs) and histone deacetylases (HDACs). Although direct mutations in major HATs (such as EP300 or CREBBP, encoding p300/CBP) are relatively uncommon in AML, oncogenic processes frequently misregulate these enzymes or exploit their functions.

The histone acetyltransferases p300/CBP, acetylation reader Bromodomain (BRD) proteins like BRD4, and histone deacetylases (HDACs) form an epigenetic regulatory triad in AML. p300/CBP are “writers” that acetylate histones at promoters and enhancers, creating open chromatin and docking sites for “reader” proteins such as BRD4 (Fig. 2). BRD4 binds acetyl-lysine marks via its tandem bromodomains and recruits transcriptional co-factors (eg, the P-TEFb complex) to sustain expression of leukaemia-promoting genes (Fig. 2) [70]. Conversely, HDACs remove acetyl marks, often leading to chromatin compaction and transcriptional repression. The balance of these activities governs AML transcriptional programs, with BRD4–p300/CBP complexes identified as key drivers of oncogenic gene expression in AML and potential therapeutic targets [70,71]. This molecular interplay has direct therapeutic implications. BET bromodomain inhibitors (BETi) targeting BRD4 can disrupt oncogenic transcription, but AML cells often adapt, via compensatory mechanisms. It was recently shown that releasing BRD4 from chromatin with a BETi triggers acute p300-dependent feedback that sustains critical AML genes [72]. This p300-driven compensation blunts BETi efficacy, but also exposes a vulnerability: sequential BET and p300 inhibition markedly improved leukaemia cell killing and prevented resistance in models [72].

Recent studies have illuminated specific dependencies on the histone acetylation machinery in AML. Using a differentiation-focused CRISPR screen, MYST-family acetyltransferase KAT6A (MOZ) was identified as a key mediator of myeloid differentiation block [73]. KAT6A was found to deposit H3K9ac at promoters, which is then recognized by the acetyl-lysine reader ENL. ENL in turn recruits additional chromatin complexes to stimulate transcriptional elongation of leukemic genes. Disrupting this writer-reader module has potent anti-leukemic effects: genetic or pharmacologic KAT6A inhibition in AML cells triggered differentiation and impaired proliferation in vitro, and extended survival in mouse models [73]. This work highlights how epigenetic co-operation between a histone acetyltransferase (KAT6A) and an acetyl-reader (ENL) sustains the leukaemic state

Another HAT, KAT2A (GCN5), has been shown to maintain AML cell identity through control of transcriptional synchronicity. Kat2a (essential for H3K9ac at promoters) loss in a conditional knockout murine AML model caused depletion of leukemia stem-like cells and induced differentiation [74]. Single-cell transcriptomics and chromatin profiling further revealed that KAT2A loss increases transcriptional noise – reducing the consistency of oncogenic gene expression bursts – which facilitates a state change from a self-renewal state towards differentiation [74]. Thus, KAT2A-mediated H3K9ac provides a buffering effect on gene expression, and AML cells are dependent on this for maintenance of their stem-like programmes.

However, overactivity of HDACs, leading to excessive deacetylation and transcriptional repression of differentiation genes, has also been observed in AML cells [75]. These data provide a rationale for using HDAC inhibitors to tilt the balance back toward a pro-differentiation state. Early trials of HDAC inhibitors (such as vorinostat and panobinostat) showed limited single-agent activity but hinted at possible synergy in combination with other treatments [76,77]. To date, no HDAC inhibitor has been shown to improve clinically meaningful AML outcomes, reflecting the need for a better understanding of patient subgroups that might benefit from this approach or improved targeting to leukemia-specific chromatin contexts.

#### The cohesin complex and 3D Genome topology in chromatin deregulation

Other chromatin-modifying genes recurrently mutated in AML include the cohesin complex [78]. The cohesin complex (eg STAG2, SMC1A, SMC3, RAD21) helps regulate 3-D genome architecture and enhancer–promoter interactions [6]. Cohesin mutations are seen in ~10%-15% of AML and alter gene expression by perturbing chromatin looping and cis-regulatory element insulation, thus facilitating or preventing enhancer-promoter communication (Fig. 2) [79]. The downstream effect is usually a subtle rewiring of transcriptional programmes that, in concert with other mutations, contributes to the leukemic phenotype [7].

In summary, histone modification patterns and chromatin structure are profoundly deregulated in AML. Whether through mutated histone writers/erasers (eg DOT1L co-option by MLL fusions, or ASXL1 loss affecting PRC2) or through broader chromatin architecture mutations, AML cells often enforce an abnormal epigenetic state that locks cells into an undifferentiated, proliferative phenotype. These findings reinforce an emerging view of AML as an epigenetically driven cancer: genetic mutations frequently converge on a few key pathways that govern the epigenome, suggesting that the epigenetic state is a final common denominator in leukaemogenesis. This has important implications for therapy – indeed, several of the mutations discussed (MLL fusions, ASXL1, Cohesin mutations) currently lack targeted therapies, but their downstream epigenetic effects provide clues for intervention. For example, the dependency of MLL-rearranged leukemias on DOT1L and on Menin has led to trials of inhibitors against these epigenetic co-factors.

### RNA modifications in AML

#### mRNA modifications

**Fig. 3:**
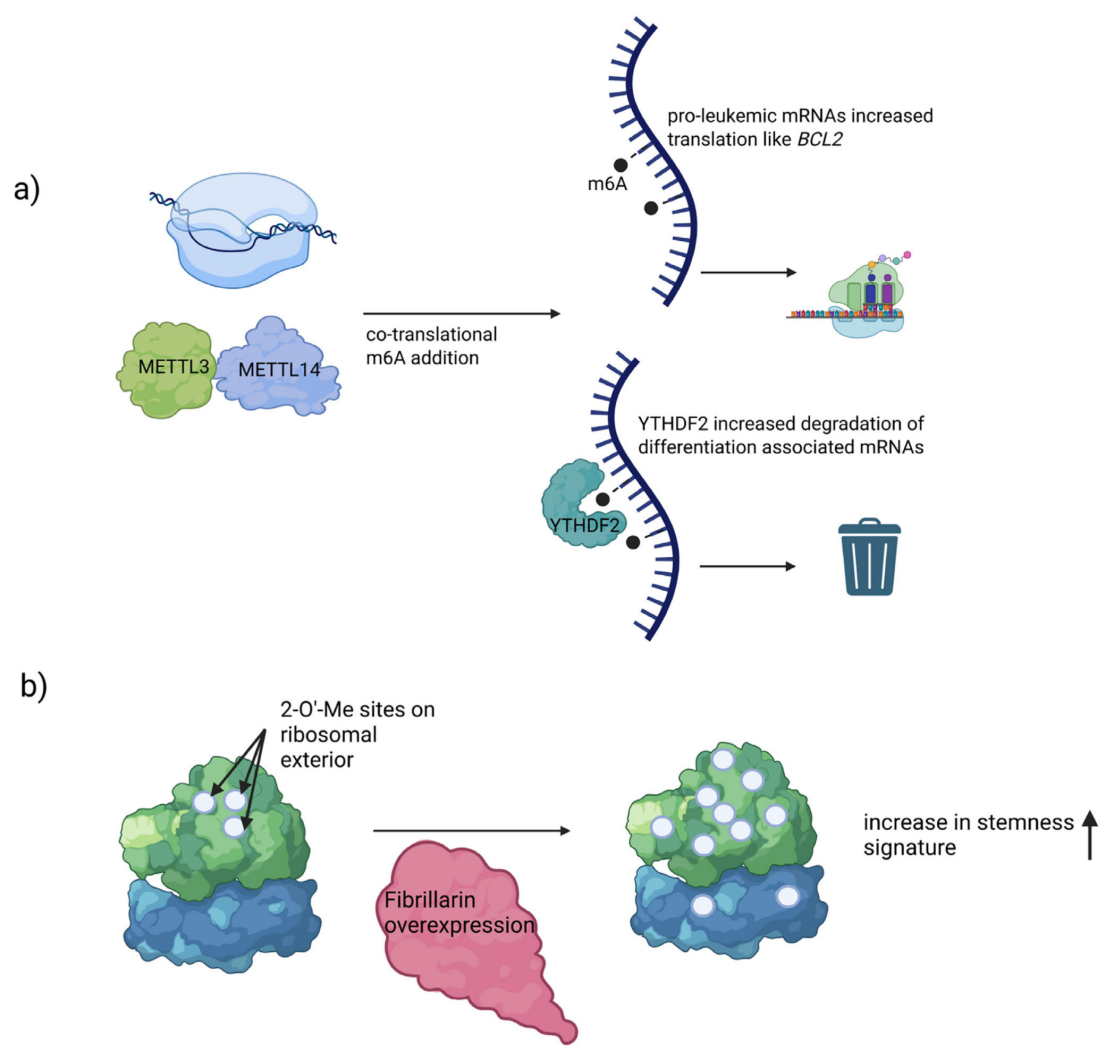
(A) Writers (METTL3–METTL14) methylate mRNA co-transcriptionally, placing m^6^A marks on key transcripts (eg, MYC, BCL2). Readers (YTHDF2) bind m^6^A-marked mRNAs that promote differentiation/apoptosis and targets them for degradation. Oncogenic/stemness mRNAs (eg, BCL2, MYC) are preferentially translated, while prodifferentiation/apoptosis mRNAs are selectively degraded, maintaining the LSC state b) Remodeled rRNA in LSCs via 2'-O-methylation. These variable methylation sites cluster on the exterior surfaces of the ribosome.Globally high rRNA 2'-O-Me levels are linked with an LSC gene-expression signature and enhanced self-renewal (stemness).

N6-methyladenosine (m6A) is the most abundant internal mRNA modification [80]. It is deposited co-transcriptionally by the METTL3–METTL14 methyltransferase complex, and removed by demethylases FTO and ALKBH5 [81,82]. In AML, emerging evidence has established m6A as a critical regulator of leukaemogenesis. METTL3 is frequently upregulated in AML and functions as a prooncogenic m6A writer. Genetic knockdown of METTL3 in AML cells causes cell-cycle arrest and induces differentiation, and METTL3-deficient leukaemia cells fail to propagate the disease in vivo. Mechanistically, METTL3 localizes to the promoters of numerous active genes and deposits m6A marks within the coding regions of their transcripts. This co-transcriptional methylation by METTL3 has been shown to enhance translation of target mRNAs, by alleviating ribosome stalling [8]. The affected transcripts include many that encode stemness or survival factors required for LSC self-renewal. For example, METTL3-mediated deposition of m6A on the BCL2 mRNA increases its stability and translation, thereby boosting BCL2 protein levels. Similarly, METTL3 promotes efficient translation of oncogenic transcriptional regulators (eg SP1 and c-MYC) that drive the stem-cell programme in AML (Fig. 3) [8]. It could be shown that METTL3 inhibition causes selective leukaemia cell lethality and prolonged survival in multiple AML models, where it led to depletion of LSC-enriched cell fractions in vitro and in vivo, indicating that AML stem cells are particularly vulnerable to loss of m6A methylation activity [83]. Through these effects, METTL3 helps sustain a gene expression programme that maintains LSCs in an undifferentiated, proliferative state.

The fate of m6A-marked mRNAs is determined by reader proteins that recognize this modification. In AML, the cytoplasmic reader YTHDF2 is highly expressed and plays a complementary role to METTL3. YTHDF2 binds to m6A-marked transcripts and recruits RNA decay complexes, reducing their stability (Fig. 3). In LSCs, this activity eliminates transcripts that would otherwise induce differentiation or apoptosis, thereby preserving the stemlike state [84]. Thus, METTL3 and YTHDF2 cooperate to enforce a leukemia-favourable transcriptome: METTL3 writes m6A marks on key growth/self-renewal transcripts to facilitate their expression, while YTHDF2 “reads” m6A on differentiation-inducing transcripts to target them for degradation, collectively preserving the LSC phenotype. The essential role of METTL3 in AML has made it a therapeutic target for eradicating LSCs. Building on these findings, STC-15, an orally bioavailable METTL3 has now progressed into clinical development.

#### rRNA modifications

Apart from mRNA methylation, the ribosomal RNA (rRNA) of AML cells – especially LSCs – can be remodelled, via specific modifications, to alter the cell’s translational program [9]. A recent study mapping 2'-O-methylation (2'-O-Me) sites on rRNAs in primary AML samples revealed a distinctive ribomethylation signature associated with the stem cell state (Fig. 3) [9]. Out of >100 known 2'-O-Me sites on rRNA, a subset showed dynamic variability in AML, with higher methylation at certain positions correlating with less differentiated, stem-like leukemic cells. These dynamically modified sites were enriched on the exterior surfaces of the ribosome (. Consistently, patient samples with globally high rRNA 2'-O-methylation at these dynamic sites were enriched for LSC gene-expression signatures, whereas more mature AML samples showed lower methylation levels [9]. Thus, enhanced rRNA 2'-O-methylation appears to drive a pro-stemness translational program in AML [9]. Indeed, forced overexpression of fibrillarin (FBL), the methyltransferase that catalyzes rRNA 2'-O-Me, was sufficient to increase overall rRNA methylation and confer stem-cell properties on non-LSC leukemia cells.

In summary, epitranscriptomic RNA modification of both mRNAs and rRNAs cooperate to sustain leukaemic stem cells. Disrupting these modifications – through inhibitors of m6A writers/readers or of rRNA hyper-methylation – is therefore a promising strategy to selectively eliminate the LSC reservoir and prevent AML relapse.

### Emerging technologies in epigenetic profiling of AML

Recent advances in sequencing technology are transforming our understanding of epigenetic heterogeneity in AML. Single-cell epigenomic profiling methods now allow investigators to map chromatin states and DNA methylation in individual leukemia cells, overcoming the averaging effect of bulk assays. For example, single-cell ATAC-seq (assay for transposase-accessible chromatin) has enabled the identification of key regulatory elements and transcription factors that govern normal haematopoietic lineage decisions, and demonstrated how these are disrupted in AML [85].

Similarly, high-resolution single-cell mapping of histone modifications via CUT&Tag and CUT&RUN is unveiling cell-to-cell variability in chromatin landscapes. In an MLL-rearranged mixed phenotype acute leukaemia cell lines and patient samples, single-cell CUT&Tag revealed that individual leukaemic cells can harbour different chromatin states at oncogenic loci – some cells showing active H3K4me3 marks and others repressive H3K27me3 at the HOXA9/TAPT1 gene promoters – reflective of a bivalent epigenetic programme that contributes to intra-tumoural heterogeneity [86]. These single-cell approaches underscore that even individual AMLs are not uniform entities, but rather a mosaic of subclones with distinct epigenetic identities, some primed toward stem-like states and others toward differentiation.

Insights like these highlight the clinical promise of single-cell epigenomics: by mapping the epigenetic heterogeneity of AML, clinicians may better predict relapse (eg, by identifying a subclone with likely drug-resistant phenotype) and design clone-specific therapies to eradicate the leukaemia at its roots.

Long-read sequencing represents another frontier in epigenetic profiling with direct clinical applicability. Unlike short-read sequencing, long-read platforms (such as Oxford Nanopore or PacBio) can sequence native DNA and detect base modifications concurrently, thereby generating an integrated genetic and epigenetic readout from single molecules. This capability has been harnessed to profile DNA methylation patterns genome-wide in AML patients in real time. For instance, a recent study introduced a rapid work-flow for nanopore methylome sequencing that could classify AML subtypes and stratify risk within days [87]. An important advantage of long-read epigenomic data is the preservation of haplotype and chromatin context over tens of kilobases, which allows phasing of mutations with nearby methylation marks and detection of allele-specific epigenetic changes.

Such findings underscore how emerging technologies – from single-cell multi-omics to long-read sequencing – are illuminating the epigenetic complexity of AML. By resolving intratumoral heterogeneity and clonal epigenetic evolution with unprecedented detail, these tools are paving the way for epigenetically informed biomarkers and more precise interventions in AML.

## Conclusion and future directions

Epigenetic deregulation is a defining feature of acute myeloid leukaemia, intricately linked to its initiation, progression, and response to therapy. This dysfunction is so pervasive that AML can be viewed as a disease of corrupted epigenetic programming: mutations in epigenetic regulators and oncogenic fusion proteins rewire the normal chromatin landscape, leading to the inappropriate silencing of differentiation pathways and activation of self-renewal genes. As detailed in this review, key epigenetic disturbances in AML include widespread DNA hypermethylation (often driven by DNMT3A, TET2, or IDH mutations), altered histone modification patterns (due to lesions in MLL/KMT2A, ASXL1, and others), disrupted higher-order chromatin structure (from Cohesin mutations), and leukemogenic RNA modifications. Each of these changes contributes to the blockade of differentiation and the uncontrolled proliferation that characterizes AML.

The past several years have witnessed the emergence of epigenetic therapies with meaningful clinical activity for AML. Hypomethylating agents have become an important tool in managing AML, particularly for those who cannot tolerate aggressive chemotherapy. IDH1 and IDH2 inhibitors, by negating a single metabolite’s effect, can release an epigenetic brake to differentiation and induce remissions, exemplifying targeted epigenetic therapy. Newly developed Menin inhibitors are poised to address the oncogenic epigenetic program in NPM1-mutant and MLL-rearranged AML, offering a much-needed option for those patients.

Looking ahead, there are several important directions for the field. One is combination therapies: epigenetic drugs may be most powerful when used in combination, either with each other or with conventional cytotoxic and immune therapies. For instance, combining DNA methylation inhibitors with histone deacetylase inhibitors could synergistically activate silenced genes. Another direction is patient stratification: as we identify distinct epigenetic subtypes of AML (for example, “DNA methylation-high” cases, or those with specific chromatin modifier mutations), treatment can be better stratified. The integration of epigenomic profiling in clinical diagnostics may guide the choice of epigenetic therapy – an approach already employed when giving IDH inhibitors to IDH-mutant AML or considering Menin inhibitors for MLL/NPM1 cases.

In conclusion, as our knowledge of epigenetic abnormalities in AML expands, so too will our therapeutic options. Epigenetic therapies have transformed aspects of AML care and hold promise for further improving outcomes when rationally applied. The interplay of genetic and epigenetic factors in AML is complex, but this complexity identifies multiple mechanisms that can be potentially therapeutically targeted. Continued research and clinical trials will refine these approaches, but the advances to date already underscore the central message: targeting epigenetic deregulation is a powerful and essential strategy in the fight against acute myeloid leukaemia.
